# Palladium, Iridium, and Rhodium Supported Catalysts: Predictive H_2_ Chemisorption by Statistical Cuboctahedron Clusters Model

**DOI:** 10.3390/ma11050819

**Published:** 2018-05-16

**Authors:** Fabien Drault, Clément Comminges, Fabien Can, Laurence Pirault-Roy, Florence Epron, Anthony Le Valant

**Affiliations:** Institut de Chimie des Milieux et Matériaux de Poitiers (IC2MP), Université de Poitiers, UFR SFA, UMR-CNRS 7285, Bât B27, 4 rue Michel Brunet, TSA 51106, 86073 Poitiers CEDEX 9, France; Fabien.drault@univ-poitiers.fr (F.D.); fabien.can@univ-poitiers.fr (F.C.); laurence.pirault@univ-poitiers.fr (L.P.-R.); florence.epron@univ-poitiers.fr (F.E.)

**Keywords:** palladium, iridium, rhodium, H_2_ chemisorption, adsorption sites, stoichiometric factors

## Abstract

Chemisorption of hydrogen on metallic particles is often used to estimate the metal dispersion (*D*), the metal particle size (*d*), and the metallic specific surface area (*S_M_*), currently assuming a stoichiometry of one hydrogen atom *H* adsorbed per surface metal atom *M*. This assumption leads to a large error when estimating *D*, *d*, and *S_M_*, and a rigorous method is needed to tackle this problem. A model describing the statistics of the metal surface atom and site distribution on perfect cuboctahedron clusters, already developed for Pt, is applied to Pd, Ir, and Rh, using the density functional theory (DFT) calculation of the literature to determine the most favorable adsorption sites for each metal. The model predicts the *H/M* values for each metal, in the range 0–1.08 for Pd, 0–2.77 for Ir, and 0–2.31 for Rh, depending on the particle size, clearly showing that the hypothesis of *H/M* = 1 is not always confirmed. A set of equations is then given for precisely calculating *D*, *d*, and *S_M_* for each metal directly from the *H* chemisorption results determined experimentally, without any assumption about the *H/M* stoichiometry. This methodology provides a powerful tool for accurate determination of metal dispersion, metal particle size, and metallic specific surface area from chemisorption experiments.

## 1. Introduction

Metallic catalysts are involved in 80% of the industrial catalytic processes [1]. These catalysts are of great importance in various fields, such as synthesis chemistry, energy production, but also, environment processes [2,3,4,5]. Among all transition metals, noble metals (or platinum group metals), such as Pd, Ir, and Rh, are of particular interest as catalysts for large scale industrial applications. A non-exhaustive list of applications for Pd include hydrogenation [6] or Suzuki cross-coupling reactions [7]. Rh is commonly used in the preparation of catalysts for the reduction of NO_x_ in automotive applications [8], and hydrogen production by steam reforming [9]. Iridium is generally used as a catalyst for propulsion applications [10] or ring opening reactions [11]. In catalysis, the activity of catalysts is currently expressed in the literature by the turnover frequency (TOF), exhibiting the activity per active site. In catalysis by metals, the mean metal particle size and the dispersion are required to be known precisely, to determine the TOF.

The hydrogen chemisorption on noble face center cubic (fcc) metals (such as Pt, Pd, Ir, and Rh) is one of the most employed characterization techniques used to determine essential parameters in catalysis, such as metallic accessibility (dispersion), particle size, as well as metallic specific surface area, exposed [12] mostly due to its ease of implementation [13].

The principle of this technique is to quantify the amount of hydrogen atoms chemisorbed on an atom located on the metal surface (*M_S_*) according to the following reaction (R1):

(R1) $$M_{S}+\alpha H_{2}\to M_{S}{\left(H\right)}_{2\alpha }$$

where 2*α* represents the chemisorption stoichiometric factor of *H* atoms chemisorbed over the number of metal atoms located on the surface of the metallic cluster, which is defined by Equation (1):

(1) $$2\alpha =\frac{H}{M_{S}}$$

If the chemisorption stoichiometric factor 2α is known, the dispersion (*D*(%)) from H_2_ chemisorption measurements may be estimated, using the following equation (Equation (2)):

(2) $$D\left(\%\right)=\frac{1}{2\alpha }\times \frac{H}{M}\times 100=\frac{M_{S}}{M}\times 100$$

where *H/M* represents the number of chemisorbed hydrogen atoms per total metal atoms.

Provided that some assumptions are made on chemisorption stoichiometric factor *(H/M_S_)* and the nature of atomic planes exposed on the surface, the particle size (*d*(nm)) and the metallic specific surface area (*S_M_*) of noble fcc metals catalysts can be obtained [14]. The common assumption is that the values of *H/M_S_* = 1 for Pt, Pd, Ir, and Rh metals [15,16]. However, some data also report *H/M_S_* stoichiometry factor exceeding unity for Pt, Pd, Rh, and Ir supported catalysts. For instance, data compiled by Bartholomew show chemisorption stoichiometric factor (*H/M_S_*) values of 1.0–1.2 for Pt, Pd, Rh, and Ir catalysts [15] Kip et al. performed careful characterization of supported platinum, rhodium, and iridium catalysts by hydrogen chemisorption and EXAFS data analysis. They reported *H/M* ratios exceeding unity for Pt (*H/*Pt = 1.14) and Rh (*H/*Rh = 1.98), and even higher than 2 for Ir (*H*/Ir = 2.68) over highly dispersed metal catalysts supported on Al_2_O_3_ and SiO_2_ [17]. McVicker et al. reported a *H/*Ir ratio close to 2 for small particle sizes (<0.6 nm) over highly dispersed Ir catalysts on Al_2_O_3_ [18]. Krishnamurthy et al. have shown that 0.48 wt% Ir/Al_2_O_3_ catalyst adsorbed up to 2.72 hydrogen atoms per iridium atom [19].

Several explanations have been proposed for *H/M_S_* ratios higher than unity, such as (i) spillover of *H* atoms from the metal to the support [20], (ii) hydride formation [21,22], (iii) the support ionicity (with zeolite) [23] or (iv) multiple adsorption on corners and edges for small metal particles [17,24].

In a previous work [25], we demonstrated that the multiple adsorption assumption is consistent with the H_2_ chemisorption literature data for the Pt catalysts [24,26,27]. For this purpose, a model describing the statistics of the surface atoms and sites (top, bridge, hollow) on perfect cuboctahedron clusters was developed. This model allowed us to assess values of *D*(%), *d* and *S*_Pt_, assuming the most favorable adsorption sites based on DFT calculation from the literature [28]. Thus, it successfully predicted, precisely, the *H/*Pt_S_ stoichiometry, which ranges from 1 to 2 for the smallest cluster (*d*_Pt_ = 0.7 nm), and the experimental values of *D*, *d*, and *S*_Pt_ determined from H_2_ chemisorption data. A set of simple equations was provided for the accurate determination of these parameters from chemisorption experiments on Pt. This approach, based on the combination of identification and quantification of adsorption sites for a given cluster shape, is expected to be valid for other fcc metals, such as Pd, Rh, and Ir.

The aim of the present study is to confirm this assumption, describe the hydrogen chemisorption properties on *M* metals (with *M* = Pd, Rh, or Ir) and determine the stoichiometric ratios *H/M_S_* using a simple methodology (statistical model) by the same philosophy as that developed in our previous work [25]. The proposed statistical model will be confronted with the *H/M* ratios and particle size values obtained from literature data.

## 2. Model Calculation

### 2.1. Dispersion, Size, Metallic Specific Surface Area, and Adsorption Surface Sites of the Cuboctahedron Crystallite

**Figure 1:**
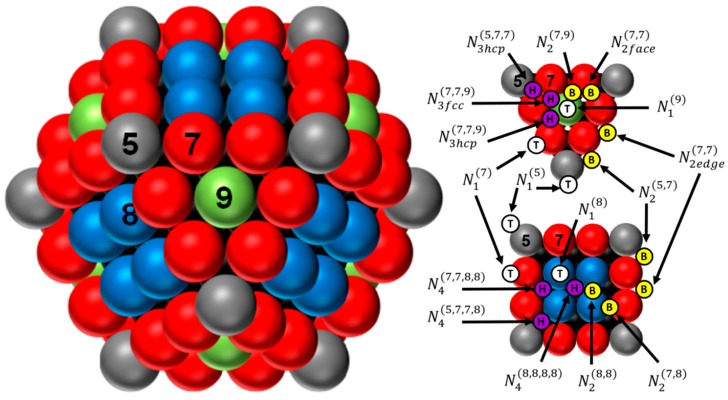
Representation of the perfect cuboctahedron (with *m* = 4) and its adsorption sites over triangular and square faces. The numbers 5 (grey), 7 (red), 8 (blue), and 9 (green) represent the coordination number of the atoms located in the corners, edges, faces (100), and faces (111), respectively. Top sites: white circle with a *T*; bridge sites: yellow circle with a B; and hollow sites: purple circle with a *H* (for more details, see ref. [25]).

The shape of Pd, Ir, or Rh crystallites (or particles) is assumed to be a perfect fcc cuboctahedron (Figure 1). This particle shape was specially chosen because it appears that the cuboctahedron shape can perfectly mimic the evolution of surface atoms of the equilibrium shape of fcc metal (icosahedron, Marks decahedron, perfect truncated decahedron and truncated octahedron) as a function of the crystallite size [25]. Using the methodologies of Van Hardeveld and Hartog [29], and our previous work [25], consisting in a systematic way of atom numbering by using mathematical series (the number of atoms are numerically counted for different cluster sizes, and a program is used to determine the logical series associated), it is possible to determine the statistics of atom distribution (*N_T_*, *N_S_*, *N_B_*, and *N_Ci_* representing the total number of atoms, surface atoms, bulk atoms, and atoms of *i* coordination number, respectively), dispersion (*D*), size (*d*), metallic specific surface area (*S_M_*), and adsorption sites (top, bridge, and hollow sites) for metal cuboctahedron cluster (Figure 1). Based on our previous work, Table 1 summarizes the enumeration and the equations giving statistics of atoms, dispersion, size, metallic specific surface area, and the number of each adsorption site for a given value of m (defined as the number of atoms lying on equivalent edge, corners atoms included, of the chosen crystallite) for Pd, Ir, and Rh metal cuboctahedron clusters, respectively [25].

### 2.2. Surface Hydrogen Adsorption Sites on Metal Cuboctahedron Crystallite (H/M) and H Chemisorption Stoichiometric Factor (H/M_S_)

For the reason of energetic considerations, hydrogen adsorption sites differ from one metal to another. Ab initio and/or DFT calculations obtained from the literature for Pd, Ir, and Rh [30,31,32,33,34,35] are therefore used to firstly determine the most favorable adsorption sites, which are evolving with the cluster size. The latter are finally used to build a unique adsorption repetitive sequence for each metal based on a linear combination of these adsorption sites to finally describe the hydrogen adsorption in the full size range. This is detailed in the following section, and summarized in Table 2. These DFT calculations generally consider pure metals, and therefore, unsupported particles, whereas nanoparticles are experimentally deposited onto a support. This raises the question about the nature of adsorption sites between supported and unsupported particles, and also, about the accessibility of a hydrogen atom over the whole metallic surface when a strong metal support interaction (SMSI) occurs. One may reasonably consider that adsorption sites are not modified by the presence of a support, since it has been demonstrated for Ir that top and bridge sites are the most favorable adsorption sites, whether the metal particle is supported [34] or not [31,33]. Next, concerning the fraction of metal interacting with the support, the metal support interaction is weakened when *H/M* ratio increases [36]. This metal support interaction weakening is the direct consequence of hydrogen insertion between the metal and the support. Therefore, the entire metal surface is accessible to hydrogen, even in the case of SMSI.

#### 2.2.1. Case of Pd

For the Pd flat surfaces, the most favorable sites for *H* adsorption are the hollow (4-fold) and the hollow (3-fold) fcc sites for Pd(100) [30] and Pd(111) [31] faces, respectively. These are representative of the large particle size domain. For the large Pd clusters, we can select $N_{4}^{\left(8,8,8,8\right)}$ adsorption sites for Pd(100), starting from *m* = 4, and $N_{3fcc}^{\left(9,9,9\right)}$ adsorption site for Pd(111), starting from *m* = 6. In the case of a smaller Pd cuboctahedron cluster (*m* = 2, 13 atoms), two stable sites for *H* adsorption were found by Watari et al. [32]. One is the hollow (4-fold) $N_{4}^{\left(5,5,5,5\right)}$ inside the square face, and the other one is the hollow (3-fold) hexagonal close packing (hcp) $N_{3hcp}^{\left(5,5,5\right)}$ of the triangular face. It has to be mentioned that these sites exist only for small particle sizes, since for *m* = 2 most of the surface atoms display a coordination number of 5. For intermediate particle size, several 4-fold adsorption sites are coexisting on the square face, which are a combination of coordination number 5 (corners), 7 (edges), and 8 (faces). This leads to two additional possibilities, which are $N_{4}^{\left(5,7,7,8\right)}$ resulting from an edge atom creation, starting from *m* = 3, and $N_{4}^{\left(7,7,8,8\right)}$ resulting from an additional face atom creation, starting from *m* = 4. In the same way, additional 3-fold hcp adsorption sites on a triangular face have to be taken into consideration as the crystallite size is increasing. These are $N_{3hcp}^{\left(5,7,7\right)}$, starting from *m* = 3 and $N_{3hcp}^{\left(7,7,9\right)}$, starting from *m* = 4. As mentioned above, 3-fold hcp sites are the most favoured for small crystallite sizes, whereas 3-fold fcc are favoured for large sizes. In this way, the additional two 3-fold hcp sites permit the transition between small and large crystallites.

Following these hypotheses, the number of *H* atoms that can be adsorbed on the Pd cuboctahedron surface (for a given m, denoted $N_{H,Pd}$) can be calculated as follows (Equation (3)):

(3) $$N_{H,Pd}=N_{3hcp}^{\left(5,5,5\right)}+N_{3hcp}^{\left(5,7,7\right)}+N_{3hcp}^{\left(7,7,9\right)}+N_{3fcc}^{\left(9,9,9\right)}+N_{4}^{\left(5,5,5,5\right)}+N_{4}^{\left(5,7,7,8\right)}+N_{4}^{\left(7,7,8,8\right)}+N_{4}^{\left(8,8,8,8\right)}$$

#### 2.2.2. Case of Ir

In the case of Ir, the most favorable sites for *H* adsorption are the bridge and the top sites for Ir(100) [33] and Ir(111) [31] faces, respectively, corresponding to the $N_{2}^{\left(8,8\right)}$ and $N_{1}^{\left(9\right)}$ adsorption sites, both starting from *m* = 4. Davis et al. calculated that the most favorable *H* adsorption sites for 38 atom truncated octahedron Ir cluster are the bridge edge sites [33], indicating that the equivalent position $N_{2}^{\left(5,7\right)}$ and $N_{2edge}^{\left(7,7\right)}$ adsorption sites have to be taken into account for small cuboctahedron clusters. Moreover, two types of adsorption sites have been suggested on the basis of DFT calculation for tetrahedron Ir_4_ cluster. These additional adsorption sites are top (corresponding to the $N_{1}^{\left(5\right)}$ adsorption site for cuboctahedron clusters) and bridge position at Ir–Ir bonds (corresponding to $N_{2}^{\left(5,5\right)}$ adsorption sites for cuboctahedron clusters) [34]. Starting from *m* = 3, an additional bridge site $N_{2}^{\left(7,8\right)}$ appears and has to be considered as another adsorption site.

According to these energetically favored adsorption sites, the number of *H* atoms that can be adsorbed on the Ir cuboctahedron surface (for a given m, denoted $N_{H,Ir}$) can be calculated as follows (Equation (4)):

(4) $$N_{H,Ir}=N_{1}^{\left(5\right)}+N_{1}^{\left(9\right)}+N_{2}^{\left(5,5\right)}+N_{2}^{\left(5,7\right)}+N_{2edge}^{\left(7,7\right)}+0.5\times N_{2}^{\left(7,8\right)}+0.5\times N_{2}^{\left(8,8\right)}$$

where the 0.5 coefficient is used to obtain a coverage of 1 monolayer with $N_{2}^{\left(7,8\right)}$ and $N_{2}^{\left(8,8\right)}$ [25].

#### 2.2.3. Case of Rh

For Rh, the most favorable sites for *H* adsorption are the hollow (4-fold) and the hollow (3-fold) fcc sites for Rh(100) [30] and Rh(111) [31] faces, respectively, corresponding to $N_{4}^{\left(8,8,8,8\right)}$ (starting from *m* = 4) and $N_{3fcc}^{\left(9,9,9\right)}$ (starting from *m* = 6) adsorption sites. DFT calculations over small sized Rh clusters (tetrahedron Rh_4_ and octahedron Rh_6_) indicated that bridge sites are the most stable [35], corresponding to $N_{2}^{\left(5,5\right)}$, for a small cuboctahedron cluster (*m* = 2). When the cluster size increases, $N_{2}^{\left(5,7\right)}$ (starting from *m* = 3) and $N_{2edge}^{\left(7,7\right)}$ (starting from *m* = 4) equivalent adsorption sites are created, due to the additional appearance of edge atoms. As shown for Pd clusters, the $N_{4}^{\left(8,8,8,8\right)}$ sites for (100) faces can lead to the creation of additional 4-fold sites ($N_{4}^{\left(5,5,5,5\right)}+N_{4}^{\left(5,7,7,8\right)}+N_{4}^{\left(7,7,8,8\right)}$) as the cluster size decreases. Finally, the number of *H* atoms that can be adsorbed on the Rh cuboctahedron surface (for a given m, denoted $N_{H,Rh}$) can be calculated as follows (Equation (5)):

(5) $$N_{H,Rh}=N_{2}^{\left(5,5\right)}+N_{2}^{\left(5,7\right)}+N_{2edge}^{\left(7,7\right)}+N_{3fcc}^{\left(9,9,9\right)}+N_{4}^{\left(5,5,5,5\right)}+N_{4}^{\left(5,7,7,8\right)}+N_{4}^{\left(7,7,8,8\right)}+N_{4}^{\left(8,8,8,8\right)}$$

#### 2.2.4. Determination of the Stoichiometric Factor and Correlation between Experimental and Model Calculations

As the number of adsorbed hydrogens as well as the total number of Pd, Ir, and Rh atoms are known, it is possible to calculate the theoretical *H/M* ratio with Equation (6).

(6) $$\frac{H}{M}=\frac{N_{H,M}}{N_{T}}$$

**Figure 2:**
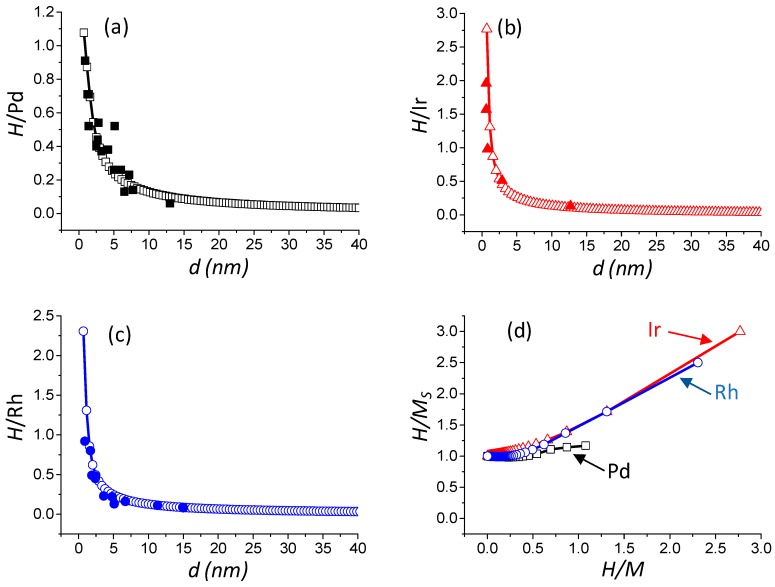
Evolution of the *H/M* ratio versus the particle size: *M* = Pd (**a**)*, M* = Ir (**b**) and *M* = Rh (**c**). Evolution of *H/M_S_* ratio versus *H/M* ratio (**d**). Full square, triangle, and circle: literature data for Pd, Ir, and Rh, respectively (see Table 3); and open square, triangle and circle: result of the statistical model calculation of this work for Pd, Ir, and Rh, respectively.

The values obtained from this statistical model have subsequently been confronted with numerous literature data [18,37,38,39,40,41,42] reported in Table 3. Results depicted in Figure 2a–c show that the model accurately predicts the literature values of *H*/Pd, *H*/Ir, and *H*/Rh, respectively. In addition, the model predicts *H/M* values in the range 0–1.08 for Pd, 0–2.77 for Ir, and 0–2.31 for Rh. The latter result clearly indicates that a single stoichiometry for Pd, Ir, and Rh cannot be used.

Knowing the *N_H,M_* value, as well as the *N_S_* number for each m value, it is possible to calculate the theoretical chemisorption stoichiometric factors with the following equation (Equation (7)):

(7) $$\frac{H}{M_{S}}=\frac{N_{H,M}}{N_{S}}$$

In order to have a representative view of the surface adsorption properties over Pd, Ir, and Rh, the *H/M_S_* theoretical chemisorption stoichiometric factors versus the theoretical *H*/*M* ratio are depicted in Figure 2d. The adsorption of one hydrogen atom per surface *M* atom (*M_S_*) is reasonably constant (near unity) for *H*/Pd < 0.54, *H*/Ir < 0.28, and *H*/Rh < 0.36, which corresponds to the large particle size domain. However, when *H*/Pd ≥ 0.44, *H*/Ir ≥ 0.28, and *H*/Rh ≥ 0.36 (small particle size domain), the *H/M_S_* ratio increases with the *H/M* ratio to reach a maximum value of 1.17, 3.00, and 2.50 for Pd, Ir, and Rh, respectively. This particular behavior directly originates from the different sites considered for hydrogen adsorption (Equations (3)–(5)), as well as their relative proportion (Table 1).

### 2.3. Determination of the Dispersion, Particle Size, and Metallic Specific Surface Area from H/M Ratios

**Figure 3:**
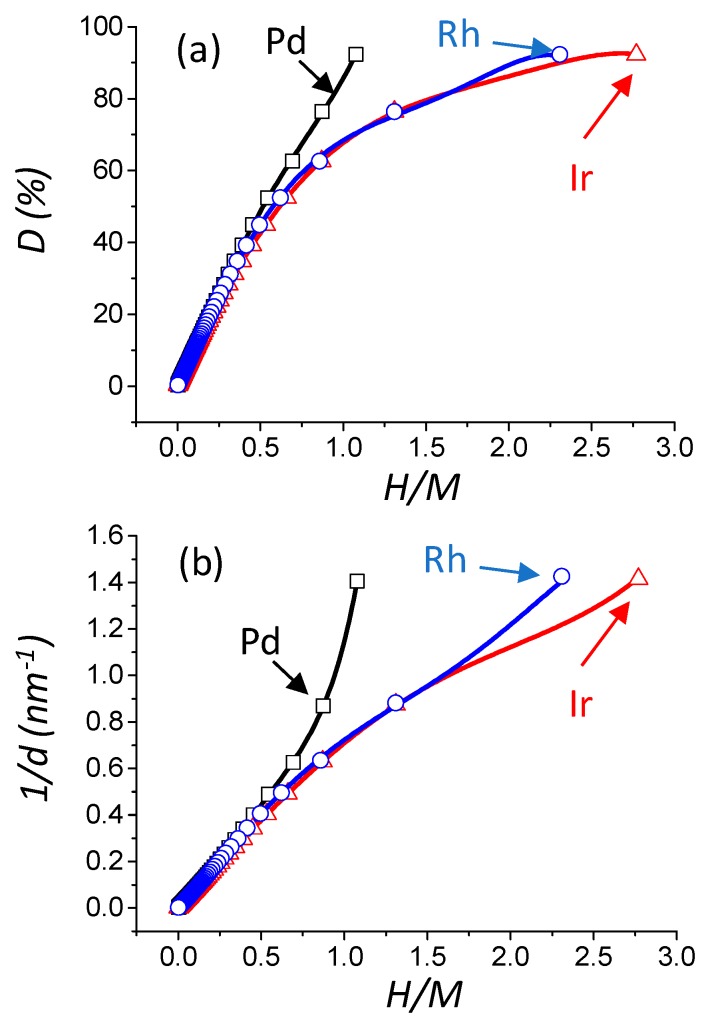

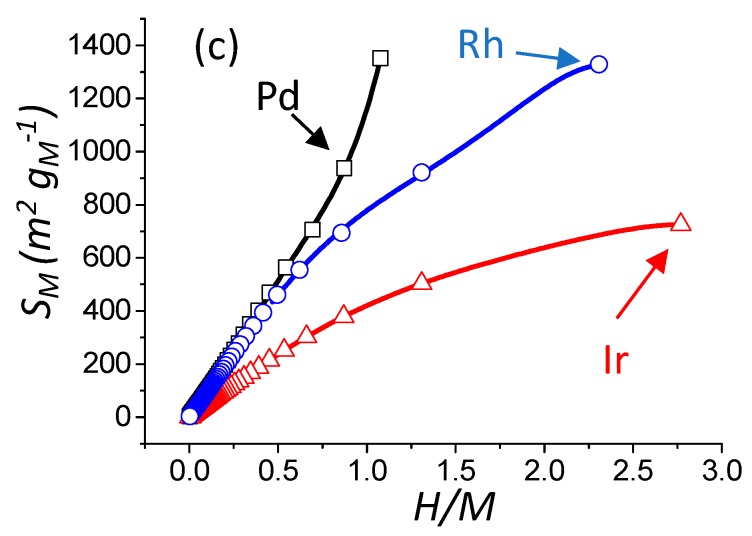
Evolution of the theoretical dispersion versus *H/M* theoretical ratio (*M* = Pd, Ir, or Rh) (**a**). Evolution of the theoretical reciprocal particle size versus *H/M* theoretical ratio (**b**). Evolution of the theoretical metallic specific surface area versus *H/M* theoretical ratio (**c**). Open square, triangle, and circle: result of the statistical model calculation of this work for Pd, Ir, and Rh, respectively. The black, blue, and red curves are the fitting result (*R^2^* = 1.000) with a 5th order polynomial trend line (see Equations (8)–(10)) for Pd, Rh, and Ir, respectively.

The knowledge of the different parameters determined by the model (*N_T_, N_S_, N_H_, D* (%), *d* (nm), and *S_M_* $\left(m^{2}g_{M}^{-1}\right)$) for any value of m allows drawing correlations with the value of *H/M* (*M* corresponding to the chosen metal), the latter being accessible from a chemisorption experiment (Figure 3a–c). It can be seen that the evolution of dispersion, particle size, as well as metallic surface area, are clearly differing from one metal to another. The physical reason for these differences lies in the different adsorption sites between Pd, Rh, and Ir. For a convenient determination of *D* (%), *d* (nm), and *S_M_* $\left(m^{2}g_{M}^{-1}\right)$, a general fifth order polynomial trend line (with the *R^2^* value equal to 1) is provided. The expression of dispersion, reciprocal particle size, and metallic surface area (see Table 1) are given below (Equations (8)–(10)), and are plotted as a function of *H/M* on Figure 3:

(8) $$D\left(\%\right)=a_{D}\times {\left(\frac{H}{M}\right)}^{5}+b_{D}\times {\left(\frac{H}{M}\right)}^{4}+c_{D}\times {\left(\frac{H}{M}\right)}^{3}+d_{D}\times {\left(\frac{H}{M}\right)}^{2}+e_{D}\times \left(\frac{H}{M}\right)$$

(9) $$\frac{1}{d}\;\left(nm^{-1}\right)=a_{1/d}\times {\left(\frac{H}{M}\right)}^{5}+b_{1/d}\times {\left(\frac{H}{M}\right)}^{4}+c_{1/d}\times {\left(\frac{H}{M}\right)}^{3}+d_{1/d}\times {\left(\frac{H}{M}\right)}^{2}+e_{1/d}\times \left(\frac{H}{M}\right)$$

(10) $$S_{M}\left(m^{2}g_{M}^{-1}\right)=a_{S_{M}}\times {\left(\frac{H}{M}\right)}^{5}+b_{S_{M}}\times {\left(\frac{H}{M}\right)}^{4}+c_{S_{M}}\times {\left(\frac{H}{M}\right)}^{3}+d_{S_{M}}\times {\left(\frac{H}{M}\right)}^{2}+e_{S_{M}}\times \left(\frac{H}{M}\right)$$

Equations (6)–(8) can be generalized by the following single equation (Equation (11)):

(11) $$Y_{M}=a_{Y}\times {\left(\frac{H}{M}\right)}^{5}+b_{Y}\times {\left(\frac{H}{M}\right)}^{4}+c_{Y}\times {\left(\frac{H}{M}\right)}^{3}+d_{Y}\times {\left(\frac{H}{M}\right)}^{2}+e_{Y}\times \left(\frac{H}{M}\right)$$

where $a_{Y},b_{Y},c_{Y},d_{Y},\;\mathrm{and}\;e_{Y}$ are constants depending on the nature of the metal *M* considered (where *M* = Pd, Rh, or Ir). The values of these empirical constants for Equation (11) are listed in Table 4.

## 3. Conclusions

The methodology described for determining stoichiometric factors for Pt clusters has been successfully generalized to 3 other fcc metals, Pd, Ir, and Rh. The use of this model clearly explains the fundamental reason for overstoichiometries experimentally observed on small particle sizes, and is related to multiple adsorption sites whose relative proportions are strongly size sensitive. The model can also be easily adapted to other shapes, provided that the surface statistics are known. The systematic use of this model for determining metallic specific surface areas from chemisorption experiments is therefore highly recommended for the accurate and meaningful calculation of turnover frequencies (TOF), which is one of the most important parameters to be determined in catalysis. We are currently investigating this aspect in our lab.

## Figures and Tables

**Table 1:** Statistics of atoms, dispersion, size, metallic specific surface area, and adsorption site numbering for metal cuboctahedron cluster. $d_{M}$ and $\rho_{M}$ represent the metallic diameter ($d_{Pd}=0.274\;\mathrm{nm}$, $d_{Rh}=0.270\;\mathrm{nm}$ and $d_{Ir}=0.272\;\mathrm{nm}$ ), and the density of the metal ($\rho_{Pd}=12.020\;{g\;\mathrm{cm}}^{-3}$, $\rho_{Rh}=12.410\;{g\;\mathrm{cm}}^{-3}$ and $\rho_{Ir}=22.562\;{g\;\mathrm{cm}}^{-3}$). $S_{C5},\;S_{C7},\;S_{C8},\;\mathrm{and}\;S_{C9}$ represent the surface area of the surface atom of type *N_C5_, N_C7_*, *N_C8_*, and *N_C9_*, respectively (for more details, see ref. [25]).

	Type	m			
		2	3	4	≥5
Atoms	$N_{T}$	13	55	147	$\frac{10}{3}\times m^{3}-5\times m^{2}+\frac{11}{3}\times m-1$
	$N_{S}$	12	42	92	$10\times m^{2}-20\times m+12$
	$N_{B}$	1	13	55	$\frac{10}{3}\times m^{3}-15\times m^{2}+\frac{71}{3}\times m-13$
	$N_{C5}$	12	12	12	12
	$N_{C7}$	0	24	48	24×(m−2)
	$N_{C8}$	0	6	24	$6\times {\left(m-2\right)}^{2}$
	$N_{C9}$	0	0	8	4×(m−2)×(m−3)
*D* (%)	Pd, Ir and Rh	92.3	76.4	62.6	$N_{S}/N_{T}\times 100$
*d* (nm)	Pd	0.7	1.2	1.6	$1.105\times {\left(N_{T}\right)}^{\frac{1}{3}}\times d_{M}$
	Ir	0.7	1.1	1.6	
	Rh	0.7	1.1	1.6	
*S_M_* (m^2^ g^−1^)	Pd	1352.2	937.3	705.7	$\frac{\left(S_{C5}+S_{C7}+S_{C8}+S_{C9}\right)\times 10^{-18}}{\frac{4}{3}\pi \times {\left(\frac{d_{M}}{2}\times 10^{-7}\right)}^{3}\times N_{T}\times \rho_{M}}$
	Ir	725.7	503.0	378.8	
	Rh	1329.1	921.3	693.7	
Top sites	$N_{1}^{\left(5\right)}$	12	12	12	12
	$N_{1}^{\left(7\right)}$	0	24	48	24×(m−2)
	$N_{1}^{\left(8\right)}$	0	6	24	$6\times {\left(m-2\right)}^{2}$
	$N_{1}^{\left(9\right)}$	0	0	8	4×(m−2)×(m−3)
Bridge sites	$N_{2}^{\left(5,5\right)}$	24	0	0	0
	$N_{2}^{\left(5,7\right)}$	0	48	48	48
	$N_{2edge}^{\left(7,7\right)}$	0	0	24	24×(m−3)
	$N_{2face}^{\left(7,7\right)}$	0	24	24	24
	$N_{2}^{\left(7,8\right)}$	0	24	48	24×(m−2)
	$N_{2}^{\left(8,8\right)}$	0	0	24	12×(m−2)×(m−3)
	$N_{2}^{\left(7,9\right)}$	0	0	48	48×(m−3)
	$N_{2}^{\left(9,9\right)}$	0	0	0	12×(m−3)×(m−4)
Hollow sites	$N_{3hcp}^{\left(5,5,5\right)}$	8	0	0	0
	$N_{3hcp}^{\left(5,7,7\right)}$	0	24	24	24
	$N_{3hcp}^{\left(7,7,7\right)}$	0	8	0	0
	$N_{3fcc}^{\left(7,7,9\right)}$	0	0	24	24
	$N_{hcp}^{\left(7,7,9\right)}$	0	0	24	24×(m−3)
	$N_{3fcc}^{\left(7,9,9\right)}$	0	0	0	24×(m−4)
	$N_{3fcc}^{\left(9,9,9\right)}$	0	0	0	4×(m−4)×(m−5)
	$N_{3hcp}^{\left(9,9,9\right)}$	0	0	0	4×(m−3)×(m−4)
	$N_{4}^{\left(5,5,5,5\right)}$	6	0	0	0
	$N_{4}^{\left(5,7,7,8\right)}$	0	24	24	24
	$N_{4}^{\left(7,7,8,8\right)}$	0	0	24	24×(m−3)
	$N_{4}^{\left(8,8,8,8\right)}$	0	0	6	$6\times {\left(m-3\right)}^{2}$

**Table 2:** Most favored hydrogen adsorption sites for Pd, Ir, and Rh flat surfaces and clusters determined from DFT/ab initio calculations.

Metal	Surface or Shape	H Adsorption Favored Sites	Ref
Pd	(100)	Hollow 4-fold	[30]
	(111)	Hollow 3-fold fcc	[31]
	Cuboctahedron (Pd_13_)	Hollow 4-fold and 3-fold hcp	[32]
Ir	(100)	Bridge	[33]
	(111)	Top	[31]
	Truncated octahedron (Ir_38_)	Bridge (edge)	[33]
	Tetrahedron (Ir_4_)	Top (corner) and Bridge (at Ir–Ir bonds)	[34]
Rh	(100)	Hollow 4-fold	[30]
	(111)	Hollow 3-fold fcc	[31]
	Tetrahedron (Rh_4_)	Bridge (edge)	[35]
	Octahedron (Rh_6_)	Bridge (edge)	[35]

**Table 3:** Literature results of H_2_ chemisorption measurements and average particle sizes (determined by TEM) for Pd, Ir, and Rh catalysts.

*M*/Support	*H/M*	*d* (nm)	Ref
Pd/SiO_2_	0.40	2.5	
Pd/SiO_2_	0.13	6.5	
Pd/Al_2_O_3_	0.41	2.5	[37]
Pd/Al_2_O_3_	0.06	13	
Pd/Al_2_O_3_	0.54	2.8	
Pd/Al_2_O_3_	0.52	1.4	
Pd/Al_2_O_3_	0.52	5.1	
Pd/Al_2_O_3_	0.14	7.7	[38]
Pd/Al_2_O_3_	0.26	6	
Pd/Al_2_O_3_	0.23	7.2	
Pd/Al_2_O_3_	0.91	0.9	[39]
Pd/Al_2_O_3_	0.26	5	
Pd/Al_2_O_3_	0.44	2.7	
Pd/Al_2_O_3_	0.37	3.2	[40]
Pd/Al_2_O_3_	0.38	4.2	
Pd/Al_2_O_3_	0.71	1.4	
Pd/Al_2_O_3_	0.71	1.2	
Ir/Al_2_O_3_	1.96	<0.6	
Ir/Al_2_O_3_	1.57	<0.6	
Ir/Al_2_O_3_	0.98	0.81	[18]
Ir/Al_2_O_3_	0.51	2.9	
Ir/Al_2_O_3_	0.13	12.7	
Rh/Al_2_O_3_	0.92	0.9	[39]
Rh/Al_2_O_3_	0.22	4.8	
Rh/Al_2_O_3_	0.80	1.7	[41]
Rh/Al_2_O_3_	0.45	2.4	
Rh/Al_2_O_3_	0.082	15	
Rh/SBA-15	0.49	1.9	
Rh/SBA-15	0.49	1.9	
Rh/SBA-15	0.48	2.4	
Rh/SBA-15	0.23	3.6	[42]
Rh/SBA-15	0.13	5.1	
Rh/SBA-15	0.16	6.7	
Rh/SBA-15	0.11	11.3	

**Table 4:** Values of the constants $a_{Y},b_{Y},c_{Y},d_{Y},\;\mathrm{and}\;e_{Y}\;$ for Equation (11). (*M*: metal; range of validity of equation 11: 0–1.08 for *H*/Pd, 0–2.31 for *H*/Rh, and 0–2.77 for *H*/Ir).

Equation	$Y_{M}=a_{Y}\times {\left(\frac{H}{M}\right)}^{5}+b_{Y}\times {\left(\frac{H}{M}\right)}^{4}+c_{Y}\times {\left(\frac{H}{M}\right)}^{3}+d_{Y}\times {\left(\frac{H}{M}\right)}^{2}+e_{Y}\times \left(\frac{H}{M}\right)$					
$Y_{M}$	M	$a_{Y}$	$b_{Y}$	$c_{Y}$	$d_{Y}$	$e_{Y}$
$D_{M}\;\left(\%\right)$	Pd	−5.055	71.208	−117.720	38.434	98.775
	Ir	−2.116	13.163	−20.633	−23.073	100.361
	Rh	−8.599	46.065	−73.064	2.015	101.969
${\left(\frac{1}{d}\right)}_{M}\;\left({\mathrm{nm}}^{-1}\right)$	Pd	1.912	−2.665	0.875	0.288	0.737
	Ir	0.000	0.038	−0.171	0.099	0.743
	Rh	−0.063	0.390	−0.771	0.414	0.753
$S_{M}\;\left(m^{2}g_{M}^{-1}\right)$	Pd	1053.493	−1139.725	−119.922	463.061	903.061
	Ir	−12.169	81.710	−176.563	39.215	487.871
	Rh	−106.053	576.518	−1005.933	413.062	900.314

## Abbreviations

d	particle size (particle diameter)
$d_{M}$	metallic diameter
$D\;\mathrm{or}\;D_{M}$	dispersion
fcc	face centered cubic
hcp	hexagonal close packing
*H*	hydrogen
$\frac{H}{M}$	number of adsorbed hydrogen per total number of metal atoms
i	coordination number
Ir	iridium
m	number of atoms lying on equivalent edge, corners atoms included
M	metal
$M_{S}$	atom on metal surface
$N_{B}$	total number of bulk atoms
$N_{Ci}$	total number of atoms of *i* coordination number
$N_{H,M}$	number of hydrogen atoms adsorbed on the metal surface
$N_{S}$	total number of surface atoms
$N_{T}$	total number of atoms
$N_{1}^{\left(i\right)}$	top adsorption site (for example $N_{1}^{\left(5\right)}$ represents the top adsorption site over a surface atom of 5 coordination number)
$N_{2}^{\left(i,i\right)}$	bridge adsorption site (for example $N_{2}^{\left(5,5\right)}$ represents the bridge adsorption site between two surface atoms of 5 coordination number)
$N_{3}^{\left(i,i,i\right)}$	hollow (3-fold) adsorption site (for example $N_{5}^{\left(5,5,5\right)}$ represents the hollow (3-fold) adsorption site between three surface atoms of 5 coordination number)
$N_{4}^{\left(i,i,i,i\right)}$	hollow (4-fold) adsorption site (for example $N_{4}^{\left(5,5,5,5\right)}$ represents the hollow (4-fold) adsorption site between four surface atoms of 5 coordination number)
Pd	palladium
Pt	platinum
Rh	rhodium
$S_{ci}$	accessible surface area of the surface atom of type $N_{Ci}$
$S_{M}$	metallic specific surface area
$\rho_{M}$	density of the metal
$\left(\frac{1}{d}\right)$	reciprocal particle size of the considered metal
$2\alpha \;or\;\frac{H}{M_{S}}$	chemisorption stoichiometric factor of hydrogen atoms over the metal surface
